# Global policy responses to the COVID-19 pandemic: proportionate adaptation and policy experimentation: a study of country policy response variation to the COVID-19 pandemic

**DOI:** 10.34172/hpp.2020.54

**Published:** 2020-11-07

**Authors:** Arlina Dewi, Achmad Nurmandi, Erna Rochmawati, Eko Priyo Purnomo, Muhammad Dimas Rizqi, Abitassha Azzahra, Samantha Benedictos, Wahdania Suardi, Dyah Tri Kusuma Dewi

**Affiliations:** ^1^Master of Hospital Administration, Universitas Muhammadiyah Yogyakarta, Indonesia; ^2^Jusuf Kalla School of Government, Universitas Muhammadiyah Yogyakarta, Indonesia; ^3^School of Nursing, Universitas Muhammadiyah Yogyakarta, Indonesia

**Keywords:** COVID-19, Pandemic, Policy

## Abstract

**Background:** Concern for the development of actions against COVID-19 has continued to grow since February 2020. Government responses remain a crucial part of preventing virus transmission through policy formulation and strengthening national capacity.

**Methods:** This study has used quantitative analysis, using secondary data from 177 countries. The variables consist of Global Health Security (GHS) category and COVID-19 pandemic. An analysis of the appropriateness of the government’s policy response in dealing with the COVID-19 pandemic was carried out by comparing the two variables.

**Results:** The study indicated a significant relationship between global health security category and pandemic score (P < 0.01). There were 37 countries out of 177 (20.9%) categorized as under-reaction and least-reaction.

**Conclusion:** Pandemic COVID-19 score, rated based on doubling time, is directly significant with the health security category. The government should improve its responsiveness and preparation to improve national capacity during the novel coronavirus pandemic.

## Introduction

After the first cases of coronavirus disease occurred in 2019 (COVID-19) in Wuhan, the capital city of Hubei province, China, the pandemic has marched relentlessly worldwide. The World Health Organization (WHO) announced COVID-19 as a global pandemic on March 11, 2020. Since then, governments worldwide have been faced with tough decisions concerning health security policies in their respective countries. How people respond to advice on preventing COVID-19 transmission is as relevant as governing.^1^ Hence, in the pandemic situation, the government plays a vital role in adapting quickly and managing the right policy to prevent pandemics from spreading quickly.

The right policy decision becomes a necessary action to prevent more impacts arising from a pandemic situation. It is because the impact of the pandemic situation has put great pressure on several aspects such as economics and social. Therefore, most countries seek to maintain or enhance their national planning capability, mainly due to lack of resources, national priorities’ conflicts, and high turnover of health workers.^2^ In an ideal world, governments should respond in a proportionate and targeted way within their operating environments.^3^

However, daily experience or governance reality can be quite different from the ideal. The response range in the COVID-19 field is often extensive, although opinions differ as to whether they are appropriate answers to a continuing problems.^4,5^ The majority of countries decide on policies about quarantine alone, although those policies may not be enough to avoid the COVID-19 spread. Therefore, the global impacts of this virus are likely to be a continuing and increasing concern for governments, policymakers, and front liners alike.^6^ The challenges faced by governments both as a whole and at the level of individual countries make it more difficult to respond.

In the COVID-19 case, as it is a new policy issue faced by the global community, political survival theory tends to have little predictive influence on policy behavior.^7^ Unlike other crises, where political survival typically motivates administrators to select a plan that includes a precautionary policy response, public health organizations follow their expert decisions and experiences to prepare against a pandemic, which can either be precautionary or proportional.^7^ The study of disproportionate policymaking is motivated by an awareness of disparities between adaptive strategies developed by individuals and the information they obtain, contributing to people’s excessive reaction to the knowledge presented.^8^ In the case of public health, studies have focused on quantifying the burden of diseases and assessing costs to determine whether a specific policy solution is proportionate to the issue or not.^1^ However, the current literature suggests that economic factors, together with public demand, focusing events, and strategic factors may lead policymakers to opt for premature or disproportionate policy actions.^5^ Today, information on the large-scale responses of countries around the world to the pandemic is minimal. It has increased the need for renewed research that targets not only the emergence of new decision-making policies but also includes the consequences of non-decisions and policy cuts.^9^ Hence, this paper can supply vital information to enhance new comparative policies among countries, particularly those formulated in responses to a global pandemic such as the current COVID-19.

Many previous studies on infectious diseases have utilized the doubling time in the measurement, such as in severe acute respiratory syndrome (SARS) and Ebola.^10,11^ However, double-time research on COVID-19, which compares the government’s response by looking at the Global Health Security (GHS), lacks. Therefore, we contribute to the available and valuable literature on comparative policy analyses by making conceptual and methodological recommendations. The disproportionate policy theory offers a useful mechanism to assess the degree of variance in transmission. We measured the government policy response level as an allegedly proportional distance from the capacity of the country’s health resources in dealing with a pandemic.

In other words, the government response can be measured and divided into several levels by comparing the double-time and GHS data in each country. We can assume that a country should be more responsive if they have a higher GHS compared to another county with lower GHS. Moreover, the degree of existence of all disproportionalities is calculated, considering domestic variances, e.g., response to crisis averages from a particular country sample. Crisis response is an action that the government must take concerning an abnormal event, the COVID-19 pandemic.

To sum up, this study aims to analyze the appropriateness of the government’s policy response in dealing with the COVID-19 pandemic by comparing each country’s health security capacities with the pandemic score.

## Materials andMethods

### Research method

**Figure 1:**
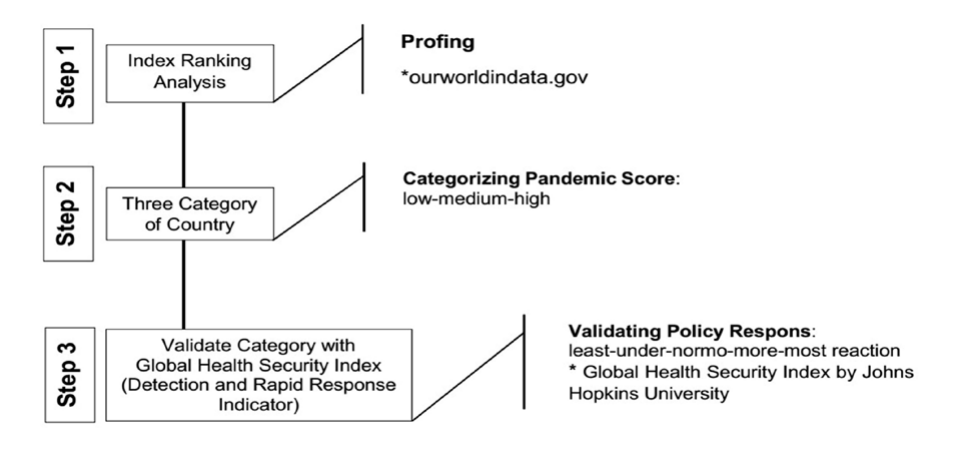
Research stages

This research is a quantitative analytic study. The initial stage begins to determine the pandemic score variable that is suitable to be associated with the global health security variable. The following steps were taken to analyze the appropriateness of the government’s policy response in dealing with the COVID-19 pandemic by comparing the health security capacities of each country with the pandemic score. There are three (3) steps (Figure 1) to investigate the policy responses of each country. For the first step, researchers mapped country reports on COVID-19 as of April 29, 2020, data “ourworldindata” database.^12^

Secondly, all countries were categorized into three clusters based on their doubling time rank. A high doubling time value indicated that the infection growth was slow in that particular country; hence, it would be categorized under ‘low pandemic’. Therefore, for the Pandemic indicator, we categorized and grouped the countries into three groups: low, medium, and high.

For the third step, we tried to test the three groups with the GHS Index, particularly with Detection and Rapid Response Indicator formulated by John Hopkins University.^13^ The GHS goal is to help understand and measure developments in global-scale capability to detect and respond to infectious disease threats. The average scores listed on the two indicators were then calculated. The results were grouped into three categories, “least prepared” if the score <33.3, “more prepared” if the score >33.3 to 66.6, and most prepared if the score 66.6-100.

The first category is the detection score. This category was chosen because it showed early detection and reporting for potential international concern epidemics, which could spread across national or regional borders. In this category, indicators assessed laboratory systems, real-time surveillance and reporting, epidemiology workforce, and data integration between the human, animal, and environmental health sectors. There were 21 questions with a total score between 0-100.

The second category is the rapid response score. Rapid response assessed emergency preparedness and response planning, exercising response plan, emergency response operation, public health and security authorities’ linking, risk communication, access to communications infrastructure, and trade and travel restrictions. There were 22 questions with a total score between 0-100.

We postulated that the countries categorized as the ‘most-prepared’ should have a good response to the pandemic compared to the countries categorized as ‘more-prepared’ or ‘least-prepared.’ However, if the countries categorized as the ‘most prepared’ had a high pandemic score, their policy response should be under or ‘least-reaction’ group. On the other hand, when the countries categorized as least prepared had a low pandemic score, they would have a ‘more’ or ‘most’ reaction policy response.

### Designing indicator of COVID-19 pandemic policy response

We used COVID-19 pandemic data that showed the infection growth rate. The data chosen for this study would encourage the policy response to take essential steps to reduce infection growth. The COVID-19 case number growth in a country showed a higher COVID-19 transmission. However, this figure was very dependent on the number of tests conducted in the country. Testing was our window into monitoring a pandemic, and at what rate it was spreading. However, if a country has completed a few tests in proportion to its population to establish a COVID-19 diagnosis, then the infection case number and the death number due to COVID-19 would be falsely low. To reduce this bias, we employed Doubling Time for growth rates. Doubling times were utilized in the study because it could measure the disease spread rate, particularly infectious disease, and indicate the magnitude of the control efforts required to curtail the diseases’ spread. Many previous studies on infectious diseases have utilized doubling time in the measurement, such as in SARS and Ebola.^10,11^ The doubling time can inform the interventions’ impact on epidemic growth, which means that doubling time changes reflect policy effectiveness.^14^

In the public health, doubling time refers to the amount of time taken to double the size or volume of a specific quantity at a constant growth rate with a formula. The main source of this data we use data from “ourworldindata.gov” (with titled: *“Global comparison: where are confirmed cases increasing most rapidly?* ”) and this web uses data sources from ECDC.^12^

To assess the relationship between global health security category and doubling time score, one-way ANOVA test was used using Jamovi 1.1.9 software.

## Results

### Analysis of the difference test between the doubling time and the global health security index category

As of April 29, 2020, the COVID-19 coronavirus affected 210 countries and territories worldwide and two international conveyances, with 1 964 845 patients currently infected. Of the 210 countries, this study only selected countries with Global Health Security data and doubling time, coming to 177 countries. The average overall health security indicator was 42.28 out of a possible score of 100. One hundred and forty-four countries did not score above 50. Table 1 shows that only 12 of 177 countries (<10%) had the most prepared Health security index category. The difference test analysis between doubling time and the global health security category showed a significant relationship (*P* < 0.01) (Table 2).

### Countries with cases of fast doubling time (highest pandemic score)

**Figure 2:**
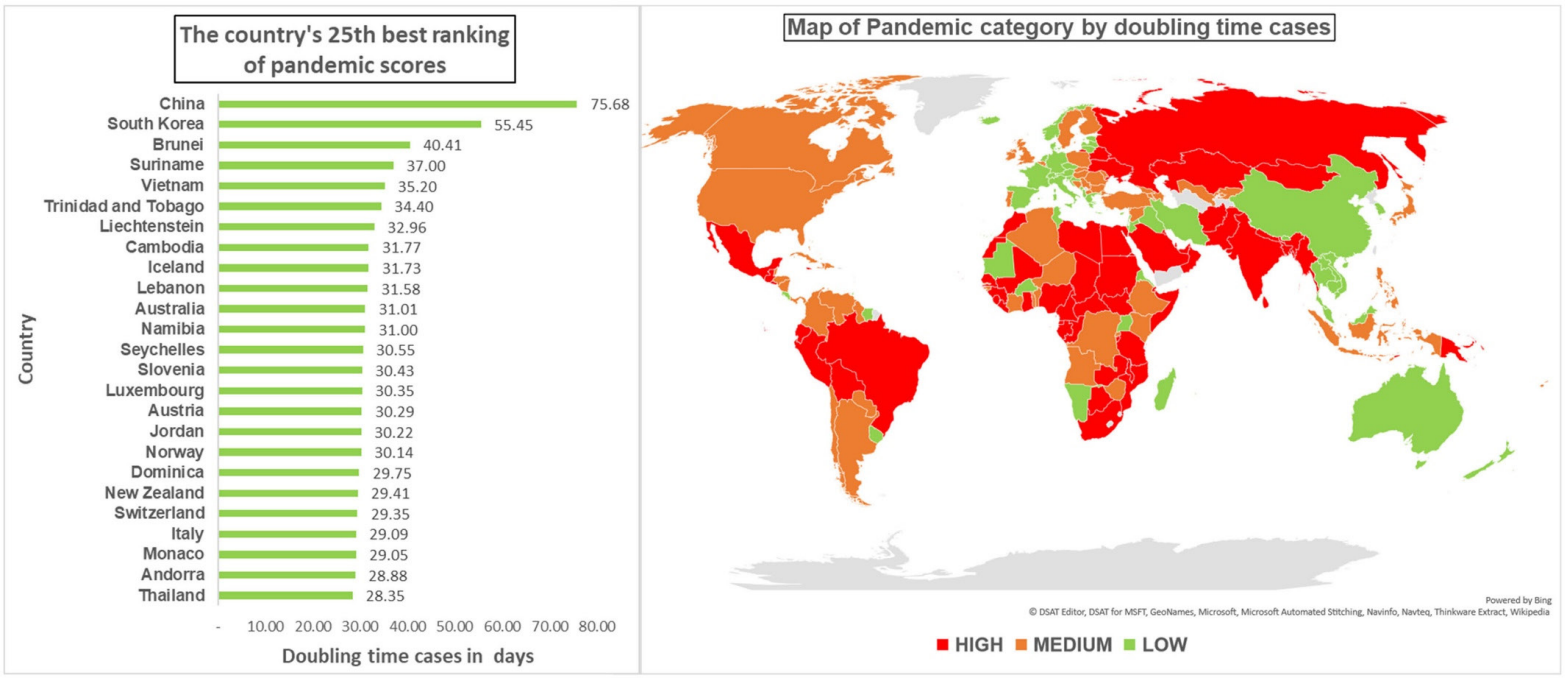
Pandemic COVID-19 score by doubling time case (25^th^ best rank and map category)

There were 59 out of 177 countries (33.3%) with a fast doubling time case (high category pandemic score). China, South Korea, and Brunei were the three countries with the best pandemic scores (Figure 2).

### Countries that fall into the most prepared category

**Figure 3:**
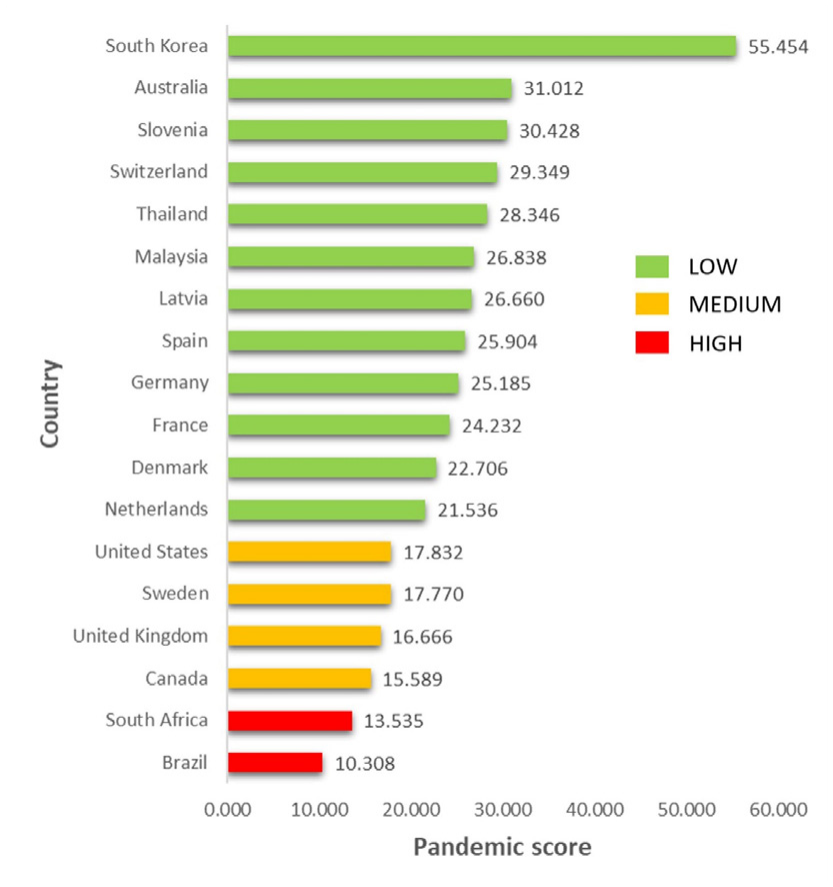
Countries with GHS categories are most-prepared compared to pandemic score

Eighteen countries were included in the most prepared category. However, six of them had a doubling time, which lied below the countries’ average in the lower category (more prepared). The countries were the US, Sweden, the UK, Canada, South Africa, and Brazil. The top five countries with the best pandemic score and a proportional response (norm action) were South Korea, Australia, Slovenia, Switzerland, and Thailand, respectively (Figure 3).

There were 12 countries with the GHS indicator of most prepared. Still, the Pandemic score was high and medium, namely Brazil, South Africa, the US, Sweden, the UK, and Canada. It indicated that the Pandemic Policy countries had under and least-reaction responses. On the other hand, 16 countries where the GHS indicator was least prepared, yet the Pandemic score was low. It signified that these countries had most-reaction (Table 3).

### Map of policy responses by country

**Figure 4:**
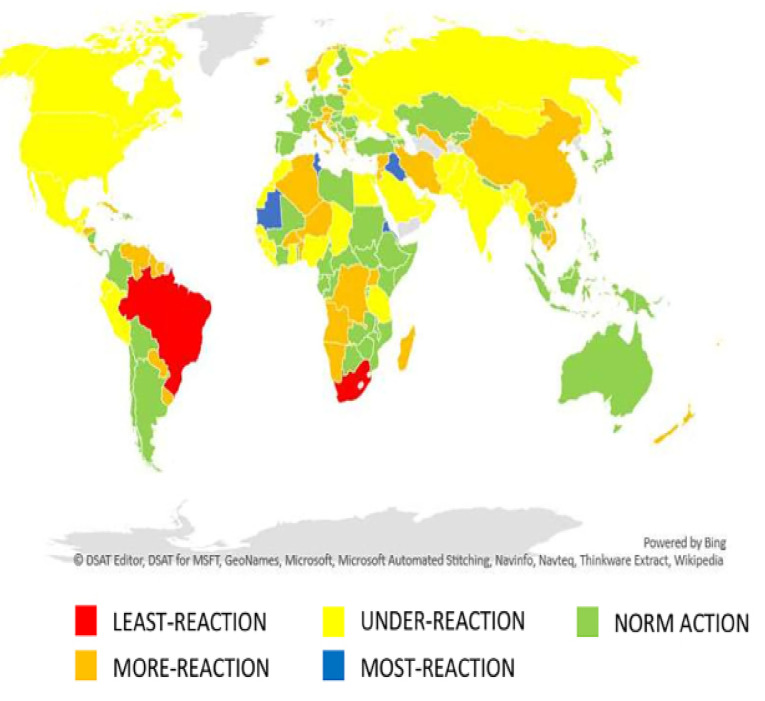
Map of policy response by country

There were 102 countries out of 177 (57.63%) with disproportional policy responses, 37 countries categorized as under-reaction and least-reaction, while 65 countries categorized as more-reaction and most-reaction (Figure 4).

## Discussion

This study found six countries categorized as most prepared in GHS indicators, but apparently, they scored medium and high in the pandemic score. Therefore, they were categorized as under-reaction and least-reaction in the pandemic policy response. The four countries included in the under-reaction category were the US, Sweden, the UK, and Canada. In contrast, the countries categorized as least-reaction were South Africa and Brazil.

Based on GHS data, the two countries in the least-reaction category scored zero in one of rapid response subcategories, which is exercising response plan. This subcategory assessed International Health Regulations (IHR) simulation exercises done by a country. When a country scored zero in this subcategory, it means that the country has not completed a biological threat-focused IHR exercise with the WHO in the past year (excluding chemical and radiological exercises). Besides, there was no evidence that in the past year, the country has undergone an exercise to identify a list of gaps and best practices through either an after-action review (post-emergency response) or a biological threat-focused IHR exercise with the WHO. Therefore, these countries might not have enough effort to build response plans to prevent and control COVID-19 outbreaks.

In South Africa, the death rate due to COVID-19 infection reached 1568 per thousand people and confirmed cases of 84.2 per thousand people. This data was taken on April 29, 2020, and the first confirmed case on March 5, 2020,^15^ was the patient being a South African returning from Italy. On March 15, the South Africa President declared a national state of disaster and announced pandemic prevention through immediate travel restrictions.^16^ Besides, the government made a different policy at the beginning of the pandemic, when other countries struggled to treat patients infected with COVID-19, the lockdown policy. It aimed to prevent the infection spread so that the health care system was not inundated with COVID-19 cases.^17^ However, the government must also consider the other side of the lockdown impact, such as the risk of deteriorating mental health and South African people’s welfare.

Communication campaigns were strengthened based on the WHO recommendations to promote awareness for healthcare professionals and the general public through 24H dedicated hotlines.^18^ South Africa was one of the countries with the highest importation risk.^19^ WHO supported active screening procedures at airports and ensured the rapid detection of the novel coronavirus. Laboratories that could evaluate samples were significant, and the WHO helps countries improve their testing capability.^20^ However, based on the latest data (May 3, 2020), COVID-19 cases in South Africa by the National Institute for Communicable Diseases (NICD) showed cases increased to 6783 confirmed cases and 131 confirmed deaths from 245747 tests.^21^ It was possible because in Africa, the region only had two referral laboratories for testing the COVID-19 infection.^20^ Besides, the laboratory also lacked personnel trained in conducting tests, limited reagent stock, and resources’ scarcity in accordance with WHO recommendations, such as quarantine spaces both at airports and hospitals, or mechanisms and/or systems to trace confirmed cases’ contacts. There remains a high need for efforts to optimize human resources through training, accelerating test results, managing confirmed cases and contacts more rapidly and preserving strict infection control measures.^19^

The primary care provision in Africa is at the forefront in giving clear, accurate, and consistent messages on infection prevention and control in communities. Patients infected with mild symptoms can be managed at home, symptomatic treatment, and self-isolation.^22^ As the government’s right hand, the primary service provider, which is closer to the community, has a role in preventing and controlling infection through specimen examination for diagnosis.

Unlike Brazil’s case, the Brazilian Ministry of Health has decided to activate EHOC-nCoV with an alert level of 1 since January 22, 2020, despite the COVID-19 pandemic case absence. It aims to coordinate actions at the national level and advise states and municipalities on secretaries of health and the federal government, public and private health services, agencies, and companies regarding contingency plans and response measures that should be proportionate and restricted current risks.^23^ Besides, the government provides information and communication to residents on basic strategies for coping with the pandemic and targeted human resource training and broadens the coverage afforded by the Brazilian National Health System (SUS). The strategic action called “O Brasilcontacomigo” (Brazil can count on me) involves the registration and training of health workers to join the fight against COVID-19.^24^

On January 27, 2020, Brazil’s first case caused an increase in alert level 2. The Brazilian government declared that COVID-19 as a public health emergency. Hence, legislation related to quarantine law was formed to protect the community and deal with public health emergency. Strategic efforts were promoted to reduce COVID-19 transmission in the community through non-pharmacological measures, such as maintaining physical distance and quarantine. The community took the primary role in this strategy. Besides, the government evaluated to minimize the pandemic impact on the community’s economy.^25^ Based on the latest data on May 3, 2020, COVID-19 cases reached 101 147 confirmed cases, and 7025 confirmed deaths.^26^

When COVID-19 spread rapidly across Asian countries, European countries went into an alarm state and started to design containment measures. Meanwhile, South American countries reacted apathetically, delaying decisions on preventive measures, and underestimating the events’ severity.^27^

The European Union (EU) has implemented numerous strategies to tackle emerging problems with incremental population-based medication and management decisions that currently define the EU’s capacities. The ability to organize, provide, and monitor care for a particular clinical population in compliance with a population-based management objective requires strict social distancing techniques, checks for, and monitoring of the antigen virus and the antibodies, separation, and therapeutic approaches, such as modern mitigation drugs and eventually a vaccine.^28^

The Brazilian government still maintains physical distance and has not implemented lockdowns to prevent COVID-19 transmission in the community.^25^ Besides, COVID-19 test kits are commercially available in 16 devices in Brazil. The meta-analysis results of the COVID-19 test accuracy showed that test equipment in circulation in Brazil could assist in emergency testing during the COVID-19 pandemic to detect IgM and/or IgG antibodies. However, the test equipment resulted in a high false-negative test detecting SARS-CoV-2 IgM antibodies, especially in acute phase.^29^ Oliveira et al explained that the high spread of COVID-19 through community transmission made the tool not tested for people suspected of being infected with COVID-19 so that test kits were prioritized for health workers and police.^24,26^ It could affect the failure of efforts to prevent transmission in Brazil, resulting in a dramatic increase in mortality.

Each country’s capacity varies in efforts to prevent, detect, and respond to outbreaks. Half of all countries have strong operational readiness capacity, which shows that effective responses to potential health emergencies are possible, including the COVID-19 pandemic. An effective response to outbreaks is not dependent on the human resources’ availability and adequate funding but also on the ability to manage emergency logistics (handling supplies for essential products needed during emergencies).

Reactive response from a policymaker, i.e., the announcement of a nationwide lockdown—was related to positive changes in how people viewed their fellow citizens and government, and better mental well-being.^30^ Other studies have used index standardizes economic responses taken by governments’ economic response (CESI=Covid19 Economic Stimulus Index). It reported that the population’s median age, the number of hospital beds per-capita, gross domestic product (GDP) per capita, and the number of total cases were all significantly associated with the extent of countries’ economic policy responses. Besides, the non-significant finding indicated that the governments’ economic reactions were more influenced by a pandemic response (i.e., infection rate) than the economic consequences of public health measures were mitigated.^31^

Therefore, a highly integrated global world, both the WHO and IHR Agreement, has the potential to be an effective tool for crisis response worldwide and risk reduction.^32^ Rapid response indicators related to emergency response operations are still below standard. Emergency response operations can be optimized through the Centers for Disease Control and Prevention (CDC) by activating emergency operations centers for coordination as COVID-19 response efforts domicile and internationally and investigating people infected with COVID-19.^33^

Study limitations encompass the inclusion of only countries with the Global Health Security data and doubling time, which may have confounded the study findings. Although, as previously stated, similar findings in the current government’s response to COVID-19 have been described, our findings may not be generalizable across countries due to different backgrounds. Nevertheless, the current study’s findings confirm other government policy research to deal with the COVID-19 pandemic.

## Conclusion

Pandemic COVID-19 score, rated based on doubling time, was directly significant with the health security category. This study discovered that more than half of the countries in its data set of 117 countries were disproportional in the Pandemic Policy Response on facing COVID-19. Six countries were categorized as most prepared in the Global Health Security category that apparently scored medium and high in the Pandemic score. Therefore, they were categorized as under-reaction and least-reaction in the Pandemic Policy Response. Brazil and South Africa were among the countries with the most-prepared health security category, but both had rapid growth of COVID-19 infections.

## Acknowledgments

Acknowledgments are to the Jusuf Kalla Government School for providing support for this research by increasing scientific collaboration through health science and government science in understanding the global pandemic COVID-19.

## Funding

We received no funding for this research.

## Competinginterests

The authors declare that they did not compete for financial interests or personal relationships.

## Ethical approval

We performed this study based on the Helsinki declaration. Obtaining approval from the institution’s ethics review committee was not applicable as we utilized secondary data.

## Authors’contributions

AD: Conceptualization, Methodology, Writing - Review & Editing, Visualization. AN: Conceptualization, Writing - Original Draft, Supervision. ER: Writing - Review & Editing. EPP: Project administration. MDR: Data Curation. AA: Data Curation. SB: Writing - Review & Editing. WS: Resources. DTKD: Resources.

**Table 1:** Characteristic countries based on global health security categories

**Global health security category (N)**	**Pandemic indicator: categories**			**Pandemic score: doubling time in day**	
	**Low**	**Medium**	**High**	**Mean**	**SD**
Least Prepared (60)	16	18	26	16.29	9.10
More Prepared (99)	31	37	31	18.53	9.65
Most Prepared (18)	12	4	2	24.41	9.85

**Table 2:** The ANOVA analysis between pandemic score and global health security category

**Pandemic score**	**df**	**SS**	**MS**	**F-value**	***P***
Between groups	2	918.663	459.332	5.100	0.007
Within groups	174	15670.534	90.061		
Total	176	16589.198			

df, degrees of freedom; SS, sum of square; MS, mean square.

**Table 3:** Pandemic policy response

**Pandemic category**	**Indicator doubling time score**	**Global health security indicator**		
		**Most prepared**	**More prepared**	**Least prepared**
		**Dimension of disproportional pandemic policy response**		
High	Rank >66.6%	**Least-reaction**	**Under-reaction**	**Norm action**
		• 2 countries: Brazil and South Africa	• 31 countries: India, Saudi Arabia, Pakistan, Ghana, Peru, Singapore, etc	• 26 countries: Libya, Somalia, Congo, Nepal, Sudan, Cameroon, etc
Medium	Rank 33.3%-66.6%	**Under-reaction**	**Norm action**	**More-reaction**
		• 4 countries: US, Sweden, UK, and Canada	• 37 countries: Portugal, Belgium, Finlandia, Japan, Indonesia, Chile, Philippines, etc	• 18 countries: Fiji, Paraguay, Niger, Angola, Syria, Cuba, Libya, etc
Low	Rank<33.3%	**Norm action**	**More-reaction**	**Most-reaction**
		• 12 countries: South Korea, Australia, Slovenia, Switzerland, Thailand, Spain, Germany, France, Denmark, Netherland	• 31 countries: China, Vietnam, Austria Italy, Iceland, Greece, Iran, Laos, etc	• 16 countries: Monaco, Brunei, Dominica, Iraq, Barbados, Tunisia, etc
